# Case Report: Endothelial Glycocalyx Damage in Critically ill Patients With SARS-CoV-2-Related Multisystem Inflammatory Syndrome (MIS-C)

**DOI:** 10.3389/fped.2021.726949

**Published:** 2021-09-06

**Authors:** Jaime Fernández-Sarmiento, Steffanie Flórez, Laura C. Alarcón-Forero, Lina María Salazar-Peláez, Julio Garcia-Casallas, Hernando Mulett, Lorena Acevedo, Carolina Salamanca

**Affiliations:** ^1^Department of Critical Care Medicine and Pediatrics, Fundación Cardioinfantil-Instituto de Cardiología, Universidad de la Sabana, Bogotá, Colombia; ^2^Graduate School, Universidad CES, Medellín, Colombia; ^3^Department of Pharmacology and Internal Medicine, Universidad de la Sabana, Chia, Colombia

**Keywords:** COVID-19, PIMS-TS, children, inflammatory, coronary aneurysm

## Abstract

Endothelial insult and damage is one of the reported consequences of SARS-CoV-2 infection. It has been associated with severe inflammation, thrombotic phenomena and profound hypoxemia in critically ill patients. Endothelial activation leads to a loss of the endothelium's antithrombotic properties which, under normal conditions, are maintained by the endothelial glycocalyx, a carbohydrate-rich layer that covers the luminal surface of endothelial cells. In children, one of the serious forms of SARS-CoV-2 virus disease (COVID-19) is multisystem inflammatory syndrome (MIS-C). This new disease is characterized by a large inflammatory response and frequent cardiovascular, cutaneous and gastrointestinal disorders. We describe the first two cases of critically ill children with MIS-C who evidenced a large inflammatory response associated with elevated plasma and imaging biomarkers of endothelial activation and endothelial glycocalyx degradation. This microcirculation involvement in MIS-C could, at least partially, explain some of the clinical manifestations and laboratory and imaging alterations found in these patients. These findings contribute to a better understanding of this disease and suggest that medications to modulate the inflammatory response and protect or restore the endothelial glycocalyx should be considered in future studies.

## Introduction

Since December 2019, when severe acute respiratory syndrome coronavirus 2 (SARS-CoV-2) infection was described, high morbidity and mortality have been seen worldwide. In April 2020, the UK and several European nations reported the appearance of a new presentation of SARS-CoV-2 infection (1), known as “*Multisystem Inflammatory Syndrome in Children* (MIS-C)” which behaves more aggressively, causing up to ten times greater mortality than that described for coronavirus 2019 disease (COVID-19) in the pediatric population (2). A recent publication in the USA found an MIS-C mortality of 2%, while COVID-19 infection in children, in most series, is mild to moderate, with a mortality of 0.1–0.2% reported in the pediatric population (3). The pathophysiological processes associated with MIS-C are still not clearly understood.

Recently, Consiglio et al. (4) carried out an analysis of plasma proteins involved in the immune response and inflammation in patients with MIS-C, including 120 proteins specific to the inflammatory response. They sought to understand the similarities and differences between MIS-C and Kawasaki disease (KD). Children with KD evidenced a hyperinflammatory response mediated by IL-17A, with elevated arteritis and coronary disease biomarkers. Children with MIS-C, on the other hand, showed autoantibodies against the structural endothelial glycoprotein known as endoglin (which is deficient in patients with hereditary hemorrhagic telangiectasia). This glycoprotein is essential for arterial endothelial integrity, and is mainly expressed in the vascular endothelium and cardiac muscle (5). Vella et al. (6) had similar findings in hospitalized children with COVID-19 and MIS-C. This group found that the immune response in MIS-C was more similar to serious COVID-19 in adults. Elevated activation of CD8^+^ T cells was seen, including a subtype of CD8^+^ cells with a vascular “*profile*” and CX3CR1 expression suggestive of endothelial activation and damage. Autopsies on adults with severe SARS-CoV-2 infection (7, 8) and, recently, on a child with MIS-C (7), have found extensive endothelial damage with macrophage activation, capillary leakage and severe microthrombosis.

These thrombotic phenomena associated with endothelial damage may be partially explained by degradation of the endothelial glycocalyx under inflammatory conditions (9). The glycocalyx is a negatively charged carbohydrate-rich layer covering the luminal surface of all blood vessels. It is responsible for modulating the mechanotransduction of the arterial and venous systems and vascular shear stress phenomena, preventing inflammatory cell migration and modulating prothrombotic states, maintaining an antiadherent endothelial phenotype (9). In pathologies such as sepsis, trauma and diabetes, among others, endothelial glycocalyx damage has been associated with thrombosis, a greater inflammatory response and worse outcomes (10). The elevation of some plasma biomarkers such as syndecan-1, endocan, chondroitin sulfate and heparan sulfate has been reported as useful for identifying endothelial glycocalyx damage (9, 10). In patients with sepsis, the elevation of syndecan-1 and other biomarkers of vascular permeability has been found to correlate with unsatisfactory outcomes such as death, the need for renal replacement therapy and coagulation disorders (11). More recently, noninvasive incident dark field (IDF) or sidestream dark field (SDF) sublingual video microscopy (*Glycocheck*™ *System*) has allowed *in vivo* identification of microcirculation and endothelial glycocalyx damage indicators (12). The system analyzes the perfused boundary region (PBR), an inverse variable of endothelial glycocalyx dimensions in sublingual microvessels. The software automatically detects, analyzes and records the PBR according to microvascular diameter from 4 to 25 microns. In healthy subjects, a value <2.0 microns has been observed in normal controls in investigations of children and adults (13, 14). In patients with sepsis, higher values have been correlated with disease severity and mortality (12, 15). This system detects blood vessel density, red blood cell velocity and glycocalyx damage, providing an estimate of microcirculatory involvement. In studies of patients with sepsis, it has been found to be useful in detecting endothelial and glycocalyx damage, correlating the information obtained by video microscopy with outcomes such as death, organ failure, the need for renal replacement therapy and coagulation disorders (9, 12, 14, 15).

## Case Description

We recently encountered two patients with Centers for Disease Control and Prevention (CDC) criteria for MIS-C who had evidence of endothelial dysfunction, glycocalyx degradation, and altered microcirculation documented by plasma biomarkers and sublingual video microscopy (Table 1).

**Table 1:** Demographic, laboratory and echocardiographic description of patients with MIS-C and endothelial and glycocalyx involvement.

**Clinical and laboratory data^**a**^**	**MIS-C Patient1**	**MIS-C Patient 2**	**CONTROL Patient**
**Clinical characteristics**			
Age (years)	8	17	15
Sex	M	M	M
Weight (kg)	18	54	60.8
Mean arterial pressure (mmHg)	52	76	93
Heart rate (beats/min)	135	95	83
Standardized capillary refill (sec)	4	3	<2
PIM 2 (%)	32.30	28.5	0.17
PELOD-2	10	1	0
**SARS-CoV-2 tests**			
SARS-CoV2 IgM antibodies	Negative	Negative	N/D
SARS-CoV2 IgG antibodies	Positive	Positive	N/D
RT-PCR	Positive	Negative	Negative
Laboratory			
Leukocytes (cel/uL)	15,700	8,380	13,600
Lymphocytes (cel/uL)	1,770	440	920
Hemoglobin (gr/dl)	9.6	17.3	15.4
Platelets (cel/uL)	63,200	114,000	259,000
Procalcitonin (ng/ml)	22.2	3.3	0.42
C-reactive protein (mg/dl)	14.71	31.92	7.04
Ferritin (mg/dL)	> 40,000	591.07	142.7
IL-6 (pg/mL)	133.8	148.6	4.2
D-dimer (mg/L)	8.26	9.39	N/D
Pro-BNP (pg/mL)	117.7	3,917	N/D
Lactate (mmol/L)	6.65	1.03	1.42
Troponin (ng/mL)	0.04	0.16	N/D
Creatinine (mg/dL)	0.6	0.8	0.7
Syndecan (ngr/mL)*			
Syndecan-1	205.3	233.93	38.3
Syndecan-1^b^	207.6	223.93	37.2
Syndecan-1^c^	224.3	227.68	47.1
**Sublingual video microscopy**			
PBR	2.56	2.29	1.68
PBR^b^	2.25	2.25	1.56
PBR^c^	2.18	2.22	1.50
PBR^d^	2.06	2.03	1.72
Capillary density D 4-6	14.6	13.3	24
CBV%	65	47.5	62.6
MHS Dyn	1.97	1.19	2.16
**Echocardiogram**			
Coronary aneurysms	Yes	No	No
Mitral regurgitation	No	No	No
Pericardial effusion	No	No	No

a *Admission values*.

b *Data 2 h after admission*.

c *Data 6 h after admission*.

d Data 24 h after admission. *Recombinant Human Syndecan-1 protein ELISA kit - abcam®. PIM: Pediatric Index of Mortality, PELOD, Pediatric Logistic Organ Dysfunction; ProBNP, Pro-B-type natriuretic peptide; CBV%, capillary blood volume percentage; MHS Dyn, Microvascular Health Score dynamic; PBR, perfused boundary region. N/D, Not done.

**Figure 1:**
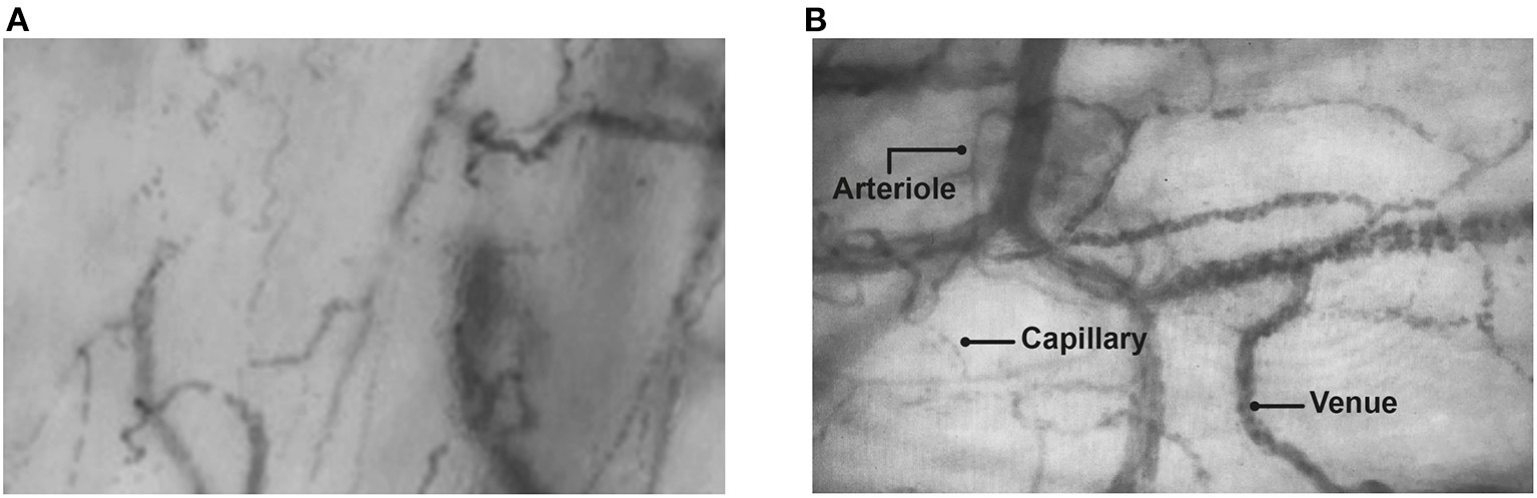
Sublingual video microscopy measurement images. **(A)** Patient 1 with MIS-C. The total number of vessels is considerably reduced and there is decreased capillary recruitment. In addition, there is a lower capillary density and smaller diameter of venules compared with the control patient. **(B)** Control patient. Normal appearance of the capillary network in the sublingual area. The capillaries, venules and arterioles are shown with normal distribution and density.

The first patient with MIS-C is an 8-year-old male with a history of living donor liver transplantation due to hepatoblastoma, who was on outpatient immunosuppression with tacrolimus and prednisolone (Table 1). He was seen in the emergency room (11/30/2020) for COVID-19 pneumonia with a positive RT-PCR. He was discharged 6 days after admission. Eight weeks later (02/01/2021), he was admitted to the intensive care unit (ICU) due to a 4-day fever associated with hypotensive shock, a maculopapular rash, respiratory distress and chest pain. The initial laboratory tests showed elevated acute phase reactants with inflammatory response, and coronary aneurysms were confirmed by echocardiogram. Serum biomarkers for endothelial glycocalyx damage (*Recombinant Human Syndecan-1 protein ELISA kit–abcam*®) were measured and sublingual video microscopy (*Glycochek*™ *System*) was performed, showing severe involvement of the endothelial glycocalyx (Figure 1A and Table 1). Figure 1A shows a reduced number of blood vessels with intermittent and slow flow in various areas (Supplementary Video 1). This lower density, perfusion heterogeneity and segmental involvement suggests profound damage to the microcirculation. This image is analyzed by the *Glycocheck*™ *System* software for calculating PBR, which was found to be very altered at the various measurement times in these patients with MIS-C, as was syndecan-1, which was measured simultaneously and was very high (Table 1). The patient required invasive mechanical ventilation and vasoactives, and 2 gr/kg of immunoglobulin were administered as well as bursts of methylprednisolone for 5 days. He was discharged after 4 weeks in the pediatric ICU (PICU). One day prior to being transferred to the hospital floor, a new measurement was taken with video microscopy and the PBR was found to have normalized (1.68). An echocardiogram was performed prior to hospital discharge, which showed persistent coronary aneurysms. Therefore, outpatient treatment with acetylsalicylic acid was ordered, as well as follow up with pediatric cardiology.

The second patient is a 17-year-old adolescent whose father had had COVID-19 5 weeks prior, and who had no other significant medical history (Table 1). He presented to the emergency room due to 5 days of headache with diarrhea and fever, abdominal pain and musculoskeletal pain. On admission, he was dehydrated and tachycardic, with abdominal pain without signs of peritoneal irritation. An RT-PCR was taken which was negative, but with positive IgG and negative IgM antibodies. An inflammatory response was seen with elevated nonspecific biomarkers such as C-reactive protein, procalcitonin and ferritin, as described in Table 1. Syndecan-1 was also elevated, and sublingual video microscopy parameters were altered. No coronary aneurysms were detected on the echocardiogram, and no mitral regurgitation or pericardial effusion were found. Fluid resuscitation was begun along with vasoactive support. Immunoglobulin was administered at 2 gr/kg along with 4 mg/kg/day of methylprednisolone for 10 days. He was discharged after 3 weeks of hospitalization. Two days prior to being transferred, the PBR was measured and found to be within the normal range (1.54). During this same period of time, a patient who underwent chest surgery for pectus excavatum correction was hospitalized in the PICU for postoperative monitoring. Inflammatory biomarkers, syndecan and sublingual video microscopy were taken as a control patient (Table 1). Figure 1B shows the normal appearance of the capillary network in the sublingual area. The capillaries, venules and arterioles can be seen. The Supplementary Video 2 shows good microvascular flow, with good capillary recruitment and microcirculation perfusion, and a normal red blood cell velocity.

## Discussion

Endothelial injury has been widely described in COVID-19, but we are unaware of reports in children showing endothelial glycocalyx damage in critically ill patients with MIS-C. Recently, in adults, Fraser et al. found biomarkers associated with endothelial glycocalyx damage, such as syndecan-1 and chondroitin sulfate, to be higher, which correlated with a prothrombotic state (16). In 19 adults with COVID-19, Rovas et al. found very elevated syndecan-1 and very reduced heparanase-2, the factor responsible for inactivating heparanase-1 which prevents endothelial glycocalyx destruction (17). They also measured the sublingual circulation and found that the endothelial glycocalyx damage indices such as PBR were very altered in adult patients who required mechanical ventilation due to SARS-CoV-2 pneumonia. These findings are consistent with the alterations we found in our two patients with MIS-C. Although PBR may be altered for other reasons such as microvascular thrombosis, inflammatory response or sepsis (9), the simultaneous elevation of syndecan-1 at the various measurement times strongly suggests endothelial glycocalyx degradation in these patients.

Endothelial glycocalyx involvement and damage in children with MIS-C could at least partially explain some of the manifestations of shock, myocardial dysfunction and cardiovascular disorders seen in these patients. As the endothelial glycocalyx degrades, the protection barrier of these cells is lost, favoring interstitial edema, capillary leakage and multiple organ failure (9). In addition, the ability to sense shear stress is lost, with the consequent release of nitric oxide from the endothelium leading to the systemic vasodilation frequently seen in children with MIS-C related shock (18). Likewise, endothelial glycocalyx damage favors the migration of inflammatory cells and elevation of acute phase reactants frequently seen in these children. Awareness of endothelial involvement and glycocalyx degradation in children with MIS-C provides a deeper understanding of the pathophysiology of this disease. It also raises the need to carry out prospective studies to determine the relationship between elevated biomarkers of endothelial glycocalyx damage or sublingual video microscopy alterations and important complications described in MIS-C such as kidney injury, multiple organ failure or death (19, 20).

In adults, SARS-CoV-2 infection is often associated with large vessel thrombosis (7, 8). There are no available descriptions of this type of events in children with MIS-C. However, since endothelial damage and glycocalyx degradation do exist, a greater risk of thrombosis could be hypothesized, given the loss of endothelial integrity and protection. In our two cases, although no large vessel thrombosis was found, there was evidence of elevated D-dimer, suggesting associated microthrombosis phenomena. Prospective studies are needed to evaluate the correlation between this endothelial damage and glycocalyx degradation, and large and small vessel thromboses in children with SARS-CoV-2 infection and MIS-C.

To our knowledge, this is the first report of endothelial glycocalyx involvement and damage in children with MIS-C. However, our report has some limitations. Only two patients with MIS-C and the experience of a single center are described. In addition, we did not measure other biomarkers that suggest increased capillary permeability (angiopoietin) or inflammatory response (interleukins), which could help further explain the pathophysiology of the problem. These cytokines are not routinely measured in middle and low-income countries. However, other serum nonspecific inflammatory biomarkers which are easily accessible in limited resource settings were measured (C-reactive protein, procalcitonin), and a significant elevation was found. Prospective studies with a greater number of patients are needed to determine if there is a correlation between the degree of endothelial damage, glycocalyx degradation and the severity of inflammation in children with MIS-C. Nonetheless, this finding, together with prior descriptions of glycocalyx alterations in adults with active SARS-CoV-2 infection (20), complements various interesting hypotheses regarding endothelial activation and damage, as well as glycocalyx degradation in these patients. These data are important because they contribute to an understanding of the pathophysiological phenomena associated with this serious disease and provide partial biological explanations for the clinical manifestations seen in critically ill patients with MIS-C.

## Data Availability Statement

The raw data supporting the conclusions of this article will be made available by the authors, without undue reservation.

## Ethics Statement

The studies involving human participants were reviewed and approved by Fundación Cardioinfantil IC. No 14569023. Written informed consent to participate in this study was provided by the participants' legal guardian/next of kin. Written informed consent was obtained from the individual(s), and minor(s)' legal guardian/next of kin, for the publication of any potentially identifiable images or data included in this article.

## Author Contributions

JF-S, SF, LA-F, LS-P, JG-C, HM, LA, and CS contributed to patient care and video microscopy measurement of microvascular damage indices, as well as writing the first draft of the manuscript. All authors contributed to manuscript revision, read, and approved the submitted version.

## Conflict of Interest

The authors declare that the research was conducted in the absence of any commercial or financial relationships that could be construed as a potential conflict of interest.

## Publisher's Note

All claims expressed in this article are solely those of the authors and do not necessarily represent those of their affiliated organizations, or those of the publisher, the editors and the reviewers. Any product that may be evaluated in this article, or claim that may be made by its manufacturer, is not guaranteed or endorsed by the publisher.

## References

[1] Jonat B Gorelik M Boneparth A Geneslaw AS Zachariah P Shah A. Multisystem Inflammatory syndrome in children associated with coronavirus disease 2019 in a children's hospital in New York City: patient characteristics and an institutional protocol for evaluation, management, and follow-up. Pediatr Crit Care Med. (2021). 22:e178–91. 10.1097/PCC.0000000000002598 33003176 PMC7924927

[2] Abrams JY Oster ME Godfred-Cato SE Bryant B Datta SD Campbell AP. Factors linked to severe outcomes in multisystem inflammatory syndrome in children (MIS-C) in the USA: a retrospective surveillance study. Lancet Child Adolesc Health. (2021) 5:323–31. 10.1016/S2352-4642(21)00050-X 33711293 PMC7943393

[3] Cui X Zhao Z Zhang T Guo W Guo W Zheng J. A systematic review and meta-analysis of children with coronavirus disease (2019). (COVID-19). J Med Virol. (2021). 93:1057–69. 10.1002/jmv.26398 32761898 PMC7436402

[4] Consiglio CR Cotugno N Sardh F Pou C Amodio D Rodriguez L. The immunology of multisystem inflammatory syndrome in children with COVID-19. Cell. (2020) 183:968–81. 10.1016/j.cell.2020.09.016 32966765 PMC7474869

[5] Schoonderwoerd MJA Goumans MTH Hawinkels LJAC. Endoglin: beyond the endothelium. Biomolecules. (2020) 10:289. 10.3390/biom10020289 32059544 PMC7072477

[6] Vella LA Giles JR Baxter AE Oldridge DA Diorio C Kuri-Cervantes L. Deep immune profiling of MIS-C demonstrates marked but transient immune activation compared to adult and pediatric COVID-19. Sci Immunol. (2021) 6:eabf7570. 10.1126/sciimmunol.abf7570 33653907 PMC8128303

[7] Ackermann M Verleden SE Kuehnel M Haverich A Welte T Laenger F. Pulmonary Vascular Endothelialitis, Thrombosis, and Angiogenesis in Covid-19. N Engl J Med. (2020) 383:120–8. 10.1056/NEJMoa2015432 32437596 PMC7412750

[8] Varga Z Flammer AJ Steiger P Haberecker M Andermatt R Zinkernagel AS. Endothelial cell infection and endotheliitis in COVID-19. Lancet. (2020) 395:1417–18. 10.1016/S0140-6736(20)30937-5 32325026 PMC7172722

[9] Fernández-Sarmiento J Salazar-Peláez LM Carcillo JA. The endothelial glycocalyx: a fundamental determinant of vascular permeability in sepsis. Pediatr Crit Care Med. (2020) 21:e291–e300. 10.1097/PCC.0000000000002266 32132499 PMC9084566

[10] Kolárová H Ambruzová B Svihálková Šindlerová L Klinke A Kubala L. Modulation of endothelial glycocalyx structure under inflammatory conditions. Mediators Inflamm. (2014) 2014:694312. 10.1155/2014/694312 24803742 PMC3997148

[11] Piotti A Novelli D Meessen JMTA Ferlicca D Coppolecchia S Marino A. Endothelial damage in septic shock patients as evidenced by circulating syndecan.1, sphingosine.1.phosphate and soluble VE.cadherin: a substudy of ALBIOS. Crit Care. (2021) 25:113. 10.1186/s13054-021-03545-1 33741039 PMC7980645

[12] Alexandros Rovas Jan Sackarnd Jan Rossaint Stefanie Kampmeier Hermann Pavenstädt Hans Vink. Identifcation of novel sublingual parameters to analyze and diagnose microvascular dysfunction in sepsis: the NOSTRADAMUS study. Crit Care. (2021) 25:112. 10.1186/s13054-021-03520-w 33741036 PMC7980588

[13] Nussbaum C Haberer A Tiefenthaller A Januszewska K Chappell D Brettner F. Perturbation of the microvascular glycocalyx and perfusion in infants after cardiopulmonary bypass. J Thorac Cardiovasc Surg. (2015) 150:1474–81. 10.1016/j.jtcvs.2015.08.050 26395044

[14] Rovas A Seidel LM Vink H Pohlkötter T Pavenstädt H Ertmer C. Association of sublingual microcirculation parameters and endothelial glycocalyx dimensions in resuscitated sepsis. Critical Care. (2019) 23:260. 10.1186/s13054-019-2542-2 31340868 PMC6657098

[15] Maitoza LA Neeman E Funaro M Pierce RW. Relevance of microvascular flow assessments in critically Ill neonates and children: a systematic review. Pediatr Crit Care Med. (2020) 21:373–84. 10.1097/PCC.0000000000002201 31834246 PMC10061570

[16] Fraser DD Patterson EK Slessarev M Gill SE Martin C Daley M. Endothelial injury and glycocalyx degradation in critically III coronavirus disease 2019 patients: implications for microvascular platelet aggregation. Crit Care Expl. (2020) 2:e0194. 10.1097/CCE.0000000000000194 32904031 PMC7449254

[17] Rovas A Osiaevi I Buscher K Sackarnd J Tepasse PR Fobker M. Microvascular dysfunction in COVID-19: the MYSTIC study. Angiogenesis. (2021) 24:145–57. 10.1007/s10456-020-09753-7 33058027 PMC7556767

[18] Perico L Benigni A Casiraghi F Ng LFP Renia L Remuzzi G. Immunity, endothelial injury and complement-induced coagulopathy in COVID-19. Nat Rev Nephrol. (2021) 17:46–64. 10.1038/s41581-020-00357-4 33077917 PMC7570423

[19] Dolhnikoff M Ferreira Ferranti J de Almeida Monteiro RA Duarte-Neto AN Soares Gomes-Gouvêa M Viu Degaspare N. SARS-CoV-2 in cardiac tissue of a child with COVID-19-related multisystem inflammatory syndrome. Lancet Child Adolesc Health. (2020) 4:790–794. 10.1016/S2352-4642(20)30257-1 32828177 PMC7440866

[20] Stahl K Gronski PA Kiyan Y Seeliger B Bertram A Pape T. Injury to the endothelial glycocalyx in critically Ill patients with COVID-19. Am J Respir Crit Care Med. (2020) 202:1178–81. 10.1164/rccm.202007-2676LE 32833500 PMC7560808

